# Modulation of the Inflammatory Response and Bone Healing

**DOI:** 10.3389/fendo.2020.00386

**Published:** 2020-06-11

**Authors:** Masahiro Maruyama, Claire Rhee, Takeshi Utsunomiya, Ning Zhang, Masaya Ueno, Zhenyu Yao, Stuart B. Goodman

**Affiliations:** ^1^Department of Orthopaedic Surgery, Stanford University, Stanford, CA, United States; ^2^Department of Bioengineering, Stanford University, Stanford, CA, United States

**Keywords:** bone healing, immunomodulation, inflammation, mesenchymal stromal cell, preconditioning, pro-inflammatory cytokines, anti-inflammatory cytokines

## Abstract

The optimal treatment for complex fractures and large bone defects is an important unsolved issue in orthopedics and related specialties. Approximately 5–10% of fractures fail to heal and develop non-unions. Bone healing can be characterized by three partially overlapping phases: the inflammatory phase, the repair phase, and the remodeling phase. Eventual healing is highly dependent on the initial inflammatory phase, which is affected by both the local and systemic responses to the injurious stimulus. Furthermore, immune cells and mesenchymal stromal cells (MSCs) participate in critical inter-cellular communication or crosstalk to modulate bone healing. Deficiencies in this inter-cellular exchange, inhibition of the natural processes of acute inflammation, and its resolution, or chronic inflammation due to a persistent adverse stimulus can lead to impaired fracture healing. Thus, an initial and optimal transient stage of acute inflammation is one of the key factors for successful, robust bone healing. Recent studies demonstrated the therapeutic potential of immunomodulation for bone healing by the preconditioning of MSCs to empower their immunosuppressive properties. Preconditioned MSCs (also known as “primed/ licensed/ activated” MSCs) are cultured first with pro-inflammatory cytokines (e.g., TNFα and IL17A) or exposed to hypoxic conditions to mimic the inflammatory environment prior to their intended application. Another approach of immunomodulation for bone healing is the resolution of inflammation with anti-inflammatory cytokines such as IL4, IL10, and IL13. In this review, we summarize the principles of inflammation and bone healing and provide an update on cellular interactions and immunomodulation for optimal bone healing.

## Introduction

The optimal treatment for complex fractures and large bone defects is an important unsolved issue in orthopedics and related specialties. In the United States alone, there are ~8 million bone fractures annually; 5–10% of fractures fail to heal and develop a non-union (1). The average cost for treatment of a bone non-union is estimated to be >US$ 10,000 (2).

Bone healing after injury is a complex biological and biomechanical process. Bone healing can be characterized by three partially overlapping phases: the inflammatory phase, the repair phase, and the remodeling phase (3). Eventual healing is highly dependent on the initial inflammatory phase, which is affected by both the local and systemic responses to the injurious stimulus. Furthermore, immune cells and mesenchymal stromal cells (MSCs) participate in critical inter-cellular communication or crosstalk to modulate bone healing. Thus, understanding and regulating inflammation is one of the key factors for successful, robust bone healing.

This review will summarize the principles of inflammation and bone healing, and provide an update on cellular interactions and immunomodulation for optimal bone healing.

## What is Inflammation?

Inflammation is the protective response of tissue to a noxious stimulus, leading to both the removal of harmful stimuli, and the initiation of the healing process (4, 5). Acute inflammation is marked by capillary dilatation and leukocytic migration and infiltration to the local area; this leads to the clinical symptoms of redness, heat, pain, and loss of function (6).

### Acute Inflammation

Acute inflammation is initiated by endogenous or exogenous adverse stimuli (4, 6). The acute inflammatory response after injury peaks within the first 24–48 h and is generally complete after 7 days (7, 8). In acute inflammation, tissue-resident cells including tissue macrophages, dendritic cells, lymphocytes, endothelial cells, fibroblasts, and mast cells recognize invading pathogens, or tissue injury byproducts, and release a variety of pro-inflammatory mediators including cytokines, chemokines, and growth factors; this results in the infiltration of polymorphonuclear neutrophils (PMNs), monocytes/macrophages, and lymphocytes into the injured site. PMNs phagocytose and eliminate invading pathogens and tissue debris. Macrophages are polarized to the M1 phenotype by damage-associated molecular patterns (DAMPs, e.g., apoptotic cells and their byproducts), pathogen-associated molecular patterns (PAMPs, e.g., bacterial endotoxin, lipopolysaccharide [LPS]), and pro-inflammatory cytokines (e.g., interferon γ [INFγ], tumor necrosis factor α [TNFα], interleukin 1β [IL1β]) (6, 9). During acute inflammation, M1 macrophages contribute to host defense as well as amplify the inflammatory reaction and recruit additional immune cells (10). M1 macrophages phagocytose and remove micro-organisms, necrotic tissue, and the provisional fibrin matrix (5, 6, 10). In addition, M1 macrophages secrete pro-inflammatory and chemotactic mediators, such as TNFα, IL1β, IL6, and C-C motif chemokine 2 (CCL2) (known as monocyte chemotactic protein 1 [MCP1]) (5, 6). These mediators initiate further recruitment of inflammatory cells and MSCs (5, 6).

### The Resolution of Inflammation

The resolution of inflammation was traditionally characterized as a passive process (11, 12). However, more recently, the resolution of inflammation is thought to be an active process, regulated by various mediators and immune cells (13).

The resolution process begins a few hours after the acute inflammatory response is initiated. First, the initial inflammatory stimuli are eliminated; subsequently, pro-inflammatory mediators are suspended, whereas anti-inflammatory (pro-resolving) mediators, such as IL4, IL10, and IL13, and CCL2, are promoted. PMNs cease to infiltrate the injury site and undergo apoptosis and subsequent efferocytosis by macrophages (14). Efferocytosis is the removal of apoptotic cells that are swiftly engulfed and digested by macrophages. Macrophages are polarized from a pro-inflammatory M1 phenotype to an anti-inflammatory tissue-repair M2 phenotype by anti-inflammatory cytokines, resulting in the advancement of the healing processes (9, 15).

Recently it has been proposed that the resolution of acute inflammation may not terminate the local immune response. Subsequent immunological activity after the resolution of the acute inflammatory stage is termed “the post-resolution stage,” to obtain a state of “adapted (or adaptive) homeostasis” at the injury site (13, 16). Adapted homeostasis alters the innate immune environment of tissues after the resolution of inflammation, including modifying the biochemical, phenotypic, and functional aspects of affected cells (13). This new adapted homeostatic environment may lead to new set-points and ranges of immune response, and is important for the maintenance of immune tolerance. This is an area of ongoing active research.

### Chronic Inflammation

Chronic inflammation is a state in which acute inflammation, fibrosis, and repair occur simultaneously (17). In chronic inflammation, monocytes/macrophages, lymphocytes, fibroblasts, and other cells are present at the injury site (17, 18). Examples of chronic inflammation include periprosthetic osteolysis due to wear particles after total joint arthroplasty, and autoimmune diseases such as rheumatoid arthritis and systemic lupus erythematosus (SLE) (9, 17). The failure of the resolution stage may lead to chronic inflammation, which may persist for prolonged periods of several weeks, or in some cases, months to years (9, 17). In chronic inflammation, adaptive homeostasis is not achieved. Continued production of pro-inflammatory cytokines such as IFNγ and TNFα continues to polarize macrophages to a pro-inflammatory M1 phenotype rather than an anti-inflammatory tissue-repair M2 phenotype. Therefore, chronic inflammation fails to establish an adaptive homeostatic state (9).

## WHAT is Bone Healing?

**Figure 1:**
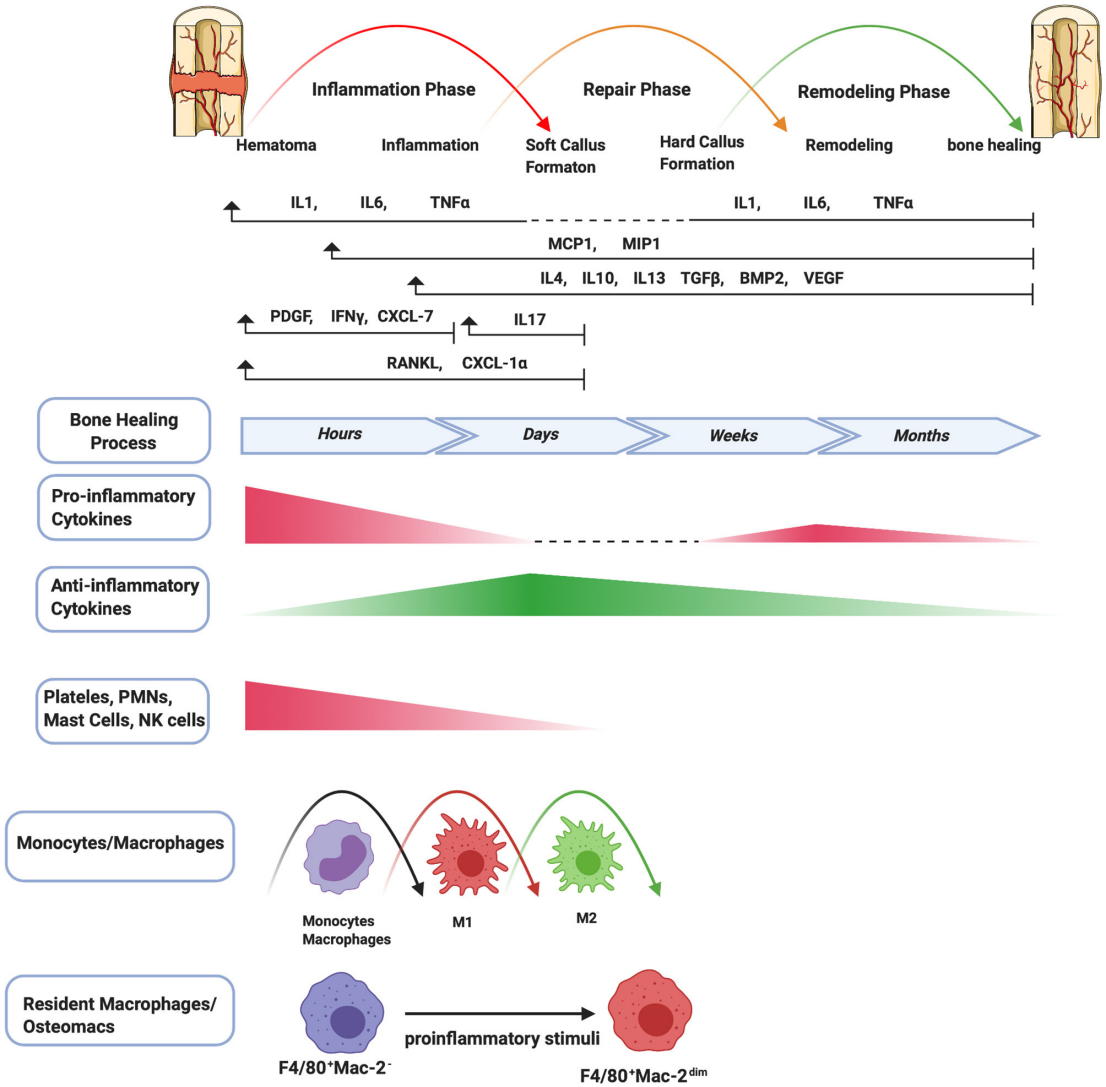
Schematic summary of the stages of bone healing and the temporal pattern of the relative immune cells and cytokines/growth factors expression. Bone healing can be viewed as a three-stage biological phase (inflammation, repair, and remodeling) which can be further divided into six main sub-steps: hematoma, inflammation, soft callus formation, hard callus formation, remodeling, bone healing. After fracture, immune cells including PMNs, NK cells, mast cells, and platelets (platelets are not truly cells as they have no nuclei) are activated in the early stage of the inflammation and the secreted cytokines/chemokines subsequently recruit and activate monocytes/macrophages to further play important roles throughout this process. The pro-inflammatory cytokines including IL1, IL6, TNFα are essential signals during the early stages of bone fracture. In addition, TNFα increases again in the late repair phase, and several pro-inflammatory cytokines (e.g., IL1, IL6, TNFα) are highly expressed in the remodeling phase. The control switch of expression patterns from a pro-inflammatory to an anti-inflammatory response (IL4, IL10, IL13) in the late stages of inflammation is critical to fracture repair.

Bone healing is an intricate regenerative process which can be classified into primary (direct) and secondary (indirect) bone healing (3, 19). Primary bone healing is uncommon in the process of fracture healing and occurs under extremely rigid fixation without any displacement of bony fragments. The fracture site is bridged by Haversian systems (or osteons), similar to the normal bone remodeling. Bone healing by Haversian systems has little or no inflammatory response and is a slow healing process that takes from a few months to a few years to achieve complete healing (3, 19). On the other hand, secondary bone healing is the most common form of fracture healing. Generally, the process of secondary bone healing is characterized by three partially overlapping phases: the inflammatory phase, the repair phase, and the remodeling phase (3) (Figure 1). Eventual healing is highly dependent on the initial inflammatory phase. Furthermore, immune cells and bone marrow-derived MSCs (BM-MSCs) participate in critical inter-cellular communication or crosstalk to modulate bone healing.

### Inflammatory Phase

An inflammatory response occurs immediately following bone fracture. The trauma leads to blood vessel rupture inside and surrounding the fracture site, resulting in a hematoma. The hematoma micro-environment is initially characterized by local hypoxia, acidity, and lower temperature, and is rich in calcium and lactic acid (20). The hematoma works as a scaffold for recruited inflammatory cells and a variety of cytokines, including IL1, IL6, TNFα, CCL2, and others, to initiate the inflammatory cascade (20). First, PMNs are recruited and then monocytes/macrophages infiltrate into the fracture site (21). Macrophages are polarized to the M1 phenotype. After infiltration of macrophages, the immune response shifts toward adaptive immunity, reflected by the invasion of lymphocytes into the fracture zone. PMNs and macrophages clear the area of dead cells and debris, and the process transforms to the resolution of inflammation, which is a complex and well-regulated activity. In this process, the agents initiating the inflammatory response and the synthesis of pro-inflammatory mediators are reduced, and the immune cells are gradually cleared from the tissue (9). Osteomacs, a special subtype of macrophages residing in bone tissues, are distributed among bone lining cells within both endosteum and periosteum and contribute bone homeostasis. Osteomacs not only sense the original injurious stimulus and initiate the inflammatory cascade, but also provide a source of molecules that begins the essential cellular events for bone healing (22, 23).

During the resolution of acute inflammation, macrophages are polarized from an M1 phenotype to an M2 phenotype by anti-inflammatory cytokines such as IL4, IL10, and IL13. BM-MSCs are attracted locally by cytokines such as TNFα (24) and stromal cell-derived factor 1 (SDF1) (known as chemokine C-X-C motif chemokine ligand 12 [CXCL12]). Recruited inflammatory cells and BM-MSCs participate in critical inter-cellular communication or crosstalk via pro-inflammatory cytokines, anti-inflammatory cytokines, as well as transforming growth factor β (TGFβ), bone morphogenetic proteins (BMPs), and growth factors (e.g., vascular endothelial growth factor [VEGF], platelet-derived growth factor [PDGF] and fibroblast growth factor-2 [FGF-2]) to initiate osteogenesis and angiogenesis (25). This process could also create a reparative granuloma forming a template for the following formation of callus (26). The acute inflammatory response peaks within 24–48 h and disappears at about 1-week post-fracture.

### Repair Phase

During the repair phase, callus is formed as vascular buds grow into the area, and the collagen matrix is laid down. Callus formation has two types of processes: intramembranous ossification and endochondral ossification (27). Intramembranous ossification occurs at the periosteum and forms hard callus directly. Periosteal MSCs differentiate into osteoprogenitor cells, which subsequently proliferate and differentiate into osteoblasts that directly form woven bone. Endochondral ossification occurs at the endosteum and bone marrow, and forms soft callus and then hard callus. BM-MSCs differentiate into chondrocytes and secrete a cartilage matrix which forms a cartilaginous template. Chondrocytes subsequently undergo hypertrophic differentiation and mineralize the surrounding matrix to form cartilaginous callus. Finally, hypertrophic chondrocytes undergo apoptosis, resulting in vascular invasion and migration of osteoblasts. Cartilage matrix subsequently coverts to bone matrix (27). Pro-inflammatory cytokines including IL1 and IL6 are absent during this phase (19). TNFα is also diminished in the early repair phase but increases in the late repair phase (24). In the endochondral ossification process, TNFα facilitates chondrocyte apoptosis, resorption of mineralized cartilage, and vascularization (24, 28). Lower levels of TNFα also enhance osteoblast proliferation but high levels inhibit these processes (24). In addition, osteomacs are enriched at sites of bone formation, forming a canopy-like structure over sites of active cuboidal osteoblasts. Osteomacs are associated with maturing of bone tissues in the repair and remodeling phases (22, 23). Alternatively, osteoclasts are highly differentiated multinucleated cells that degrade bone.

### Remodeling Phase

At the late stage of bone healing, bone is restored to its original structure, shape, and mechanical properties by remodeling. The balance between osteoblastic and osteoclastic activity which results in lamellar bone deposition and bone resorption plays an important role during the remodeling stage (19, 27). Several pro-inflammatory cytokines (e.g., IL1, IL6, and TNFα) are highly expressed (3, 19, 26).

## Is Inflammation Necessary to Obtain Bone Healing?

Inflammation is the crucial first step for bone healing as described above. However, deficiency and inhibition of acute inflammation can lead to impaired bone healing. For example, it is well-known that drugs such as non-steroidal anti-inflammatory drugs (NSAIDs), corticosteroids, chemotherapeutic agents and others increase the risk for non-union (29, 30). In addition, excess acute inflammation due to severe injuries such as polytrauma, and open fracture also increases the risk for impaired bone healing (31). Furthermore, chronic inflammation is detrimental to bone healing (31).

### Deficiency and Inhibition of Acute Inflammation

NOD/SCID-IL2Rγc^null^ mice are innate and adaptive immunodeficient mice that lack functional monocytes, dendritic cells, natural killer cells, and lymphocytes (32). In addition, there is a defect in the common gamma-chain of the IL2 receptor (γ_c_) such that signaling of IL-2, IL-4, IL-7, IL-9, IL-15, and IL-21 is defective (33). The fracture healing process in NOD/SCID-IL2Rγc^null^ mice demonstrates a callus whose bone content is unaffected during the early healing stage; however, the callus is reduced during the late healing phase and the amount of cartilage is significantly increased, indicating delayed endochondral ossification (34).

The pro-inflammatory cytokine TNFα has a complex role during bone healing, demonstrating biphasic peaks at 72 h and 3 weeks after injury (35). TNFα-receptor-deficient mice showed delayed endochondral and intramembranous bone formation (24, 36, 37). Furthermore, TNFα knockout mice demonstrated poor endochondral bone repair after fracture, but had normal skeletogenesis, implicating TNFα signaling specifically in early fracture repair (38). IL6 has been shown to increase osteoclastogenesis (39). IL6 knockout mice demonstrated delayed fracture healing, with decreased osteoclastogenesis and impaired callus formation, but showed comparable bone healing and strength in the late stage of fracture healing (40, 41). The IL6 signal has two different pathways: the classic signaling pathway via the membrane-bound receptor (mLI-6R) and the trans-signaling pathway via its soluble form (sIL6R) (42). Recent studies using a fracture model in rats demonstrated that the classic signaling pathway regulates the immune response and bone healing, whereas the trans-signaling pathway has a minor effect on the immune response and does not influence bone healing (43); rather it has negative effects on bone healing after severe trauma (44). IL17A, secreted mainly by γδ T cells, has also been characterized as an inflammatory cytokine (45). IL17A expression is induced in the early phase of bone healing and stimulates the proliferation and osteoblastic differentiation of BM-MSCs (45–47). IL17A knockout mice exhibited delayed callus formation and lower bone mineral density due to a decrease in osteoblastic bone formation (45). CCL2 is an important chemokine expressed early in inflammatory conditions. CCL2 deficient mice showed diminished infiltration of macrophages and BM-MSCs, and impaired vascularization, resulting in delayed fracture healing, and less callus formation (48). Thus, pro-inflammatory cytokines and chemokines are critical to bone healing. Conversely, although IL1β promotes the proliferation and differentiation of murine pre-osteoblasts *in vitro*, IL1β receptor knockout mice had normal bone healing (49).

Prostaglandin E2 (PGE2) plays a key role in bone metabolism including homeostasis, inflammation, and healing (50, 51). Cyclooxygenase 2 (COX-2) is a key enzyme important to PGE2 synthesis. COX-2 knockout mice exhibited reduced osteoblastogenesis and impaired intramembranous and endochondral bone healing (52). NSAIDs have analgesic, antipyretic, and anti-inflammatory effects, and are frequently used to treat musculoskeletal conditions. Continuous use of NSAIDs is associated with delayed bone healing and non-union via inhibition of the COX2-PGE2 pathway (53, 54). In our previous study, co-culture of murine BM-MSCs with undifferentiated M0, pro-inflammatory M1, or anti-inflammatory M2 macrophages showed that celecoxib, a COX-2 selective NSAID, reduced bone mineralization in all co-cultures but most dramatically in the BM-MSC-M1 co-cultures (55). This study re-emphasized the importance of an initial transient acute inflammatory period during fracture healing.

### Excess of Inflammation vs. Bone Healing

Excessive acute inflammation can be caused by several stimuli including microbial infection, surgical intervention, and injury due to mechanical, chemical, electrical, or thermal trauma (56), leading to the excessive production of pro-inflammatory cytokines (56, 57). TNFα stimulates osteoclastogenesis and inhibits osteoblast function (58). TNFα overexpression increases NFκB and the mitogen-activated protein kinases (MAPKs), increasing the release of cytokines and chemokines and leading to the activation of osteoclasts (58). In addition, TNFα inhibits BM-MSC differentiation into osteoblasts via the ubiquitin E3 ligase Wwp1 (59, 60). In polytrauma, excessive infiltration of activated neutrophils to the fracture site leads to poor fracture healing (21). Coculture of human BM-MSC with CD4+ T cells promoted increased expression of bone markers and mineralization, however, coculture of human BM-MSC with CD8+ T cells did not (61). Patients with impaired fracture healing had a lower CD4+/CD8+ ratio compared to patients with healed fractures (62). These data indicated that CD4+ T cells promoted osteogenic differentiation of human BM-MSCs. On the other hand, in patients with severe trauma, the CD4+/CD8+ ratio has been reported to be decreased (63). A rat model of tibial fracture with severe overlying muscle injury demonstrated not only excessive and prolonged infiltration of T cells but also a low CD4+/CD8+ ratio at 14 and 28 days, compared to a rat model of tibial fracture only. Furthermore, this rat model of tibial fracture with muscle injury showed persistence of M1 macrophages, resulting in impaired bone healing (64). Thus, excessive and/or prolonged acute inflammation alters the balance of inflammatory cells and inflammatory cytokines, which may lead to poor bone healing.

### Chronic Inflammation vs. Bone Healing

Chronic inflammation can be highly detrimental to bone healing. In chronic inflammation, TNFα and the NFκB signaling pathways are continuously upregulated, resulting in the differentiation and activation of osteoclasts (6, 65–68). High and persistent TNFα levels damage tissues and reduce bone volume (58). In the context of high TNFα levels, the eroded bone surface, which is a parameter of bone resorption, was significantly increased histomorphometrically in patients with inflammatory diseases, such as rheumatoid arthritis, Crohn's disease, and bronchial asthma (69). Chronically elevated NFκB activity was associated with the impaired ability of BM-MSCs to form bone (70). Chronic inflammation due to polyethylene particles induced the activation of NFκB pathways, leading to bone resorption in both the femoral intramedullary continuous polyethylene particle infusion model (71) and in the murine calvarial model (72). In addition, prolonged M1-macrophage activation continuously produced cytokines, resulting in bone resorption via increased osteoclast activity and suppression of bone formation by osteoblasts (5). Therefore, failed bone healing in chronic inflammation can be caused by the imbalance of M1/M2 macrophages (5).

## What Happens if Bone Healing Does Not Occur?

According to the U.S. Food and Drug Administration (FDA), non-union is defined as a fractured bone that has not healed after a minimum of 9 months since the injury and shows no radiographic progressive bone healing for a minimum of 3 consecutive months (73). Although different definitions have been proposed, non-unions can be radiologically categorized as hypertrophic and atrophic non-unions (74).

### Hypertrophic Non-union

On radiographs, a hypertrophic non-union is seen as a large broad callus facing the radiolucent fracture gap and is referred to as a horse-shoe or elephant-foot non-union (75). Hypertrophic non-union is believed to be caused by inadequate immobilization to maintain the necessary vascularity and biologic viability for complete bone healing to occur (76, 77). However, a previous *in vitro* study (78) demonstrated that cell viability and osteogenic ability of human osteoblasts isolated from hypertrophic non-unions were decreased. Iwakura et al. (79) showed that hypertrophic non-union tissue contained mesenchymal progenitor cells with multilineage capacity, but their proliferative capacity was decreased. Thus, the biological mechanisms and immunoregulatory controls in hypertrophic non-union remain largely unknown.

### Atrophic Non-union

Atrophic non-union refers to an inadequate or poorly vascularized mesenchymal tissue with very limited potential for successful healing of the bone ends. Radiographically, there is little callus formation around a fibrous tissue-filled fracture gap (75). An *in vitro* study demonstrated that the fibroblast-like cells isolated from atrophic non-union tissues in humans had lower cell proliferation and osteogenic ability (80). El-Jawhari et al. (81) showed that BM-MSCs isolated from the iliac crest in patients with a non-union had low proliferative capacity and osteogenic ability compared with those with successful union. In addition, the passage-zero cultured BM-MSCs treated with a mixture of IFNγ, TNFα, IL1, and IL17 showed lower gene expression of indoleamine 2,3-dioxygenase (IDO), prostaglandin E synthetase 2 (PGES2), and TGFβ1, indicating reduced immunosuppressive potential. The immunoregulatory properties of cells in atrophic non-unions have not been fully clarified.

## Can we Modulate Inflammation to Facilitate Healing?

As stated previously, an initial and optimal transient stage of acute inflammation is a crucial event during fracture healing. One approach to facilitating bone healing by means of immunomodulation is the preconditioning of MSCs to empower their immunosuppressive properties (82). Preconditioned MSCs (also known as “primed/ licensed/ activated” MSCs) are cultured first with pro-inflammatory cytokines or exposed to hypoxic conditions for a few days to mimic the inflammatory microenvironment prior to their intended application in *in vitro* and *in vivo* studies in animals and humans (e.g., differentiation into special cell lineages, loading into scaffolds, and administration to animals and humans). Another approach involves the resolution of inflammation with anti-inflammatory cytokines.

### Preconditioned MSC With Pro-Inflammatory Cytokines

**Figure 2:**
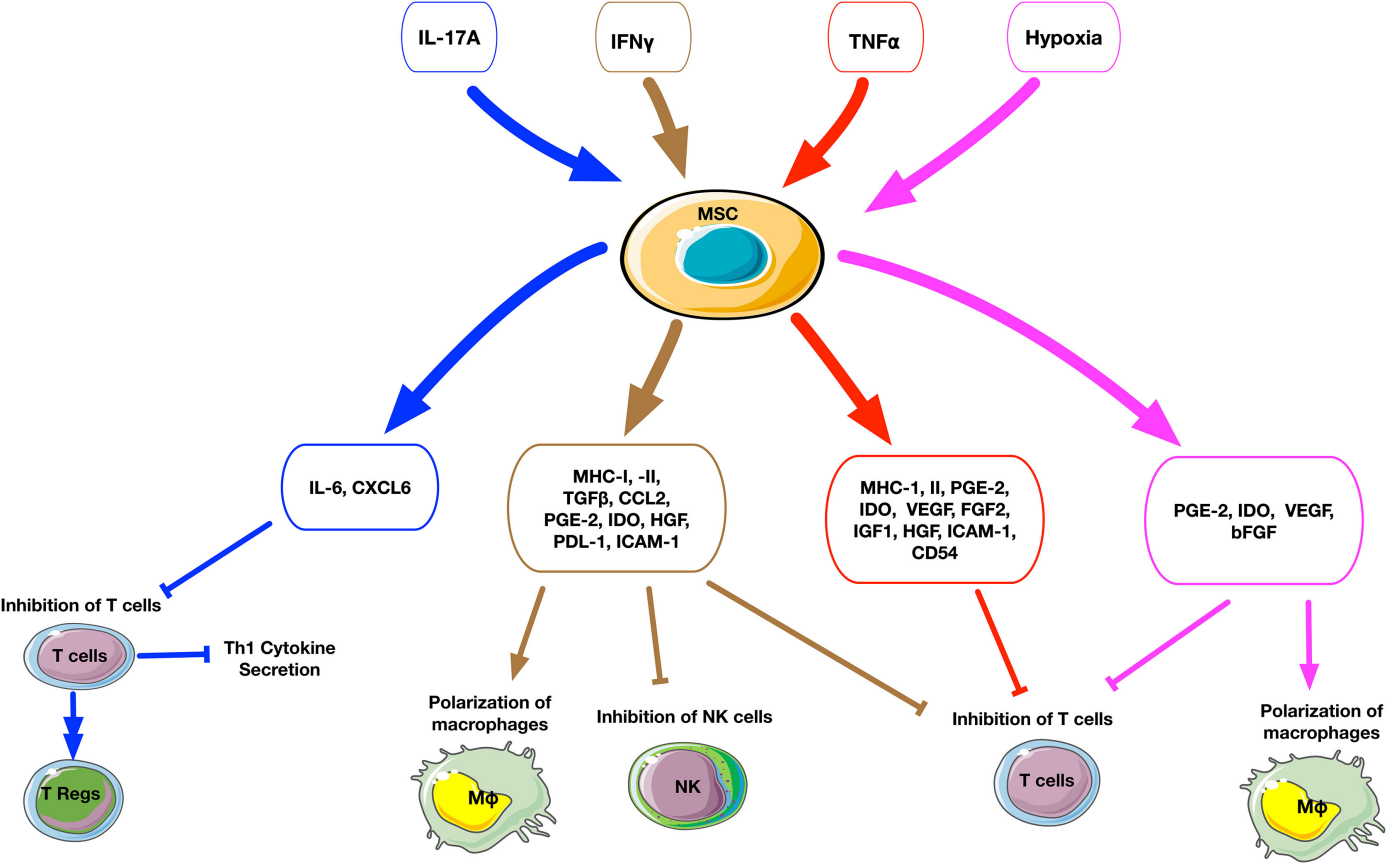
Schematic summary of the cellular and molecular effects after preconditioning of MSCs. The upper small rectangle boxes depict the different stimuli used to precondition MSCs. The middle level boxes indicate the upregulation of cytokines and chemokines by MSCs after stimulation. The effects of preconditioned MSCs on a cellular level are represented on the bottom of the figure. The stimuli factors and their respectively triggered outputs are linked by matched color arrows and boxes. IL17A-preconditioned MSCs increase IL6 secretion and regulatory T cell generation and inhibit Th1 cytokine secretion such as TNFα, IFNγ, IL2, and IL10. IFNγ-preconditioned MSCs promote the secretion of immunomodulatory molecules such as IDO, PGE2, HGF, TGFβ, and CCL2, suppress T cell and NK cell proliferation and polarize macrophages to an M2 phenotype. TNFα-preconditioned MSCs promote the secretion of immunoregulatory mediators such as PGE2, IDO, and HGF, suppress T cell proliferation. Hypoxia-preconditioned MSCs promote the secretion of PGE2, IDO, VEGF, and bFGF, suppress T cell proliferation, and polarize macrophages to an M2 phenotype.

Addition of different combinations of inflammatory cytokines to cell culture can dramatically affect the secretory profile and osteogenic ability of MSCs (Figure 2, Table 1). IFNγ-preconditioned MSCs upregulated IDO and the secretion of immunomodulatory molecules, such as PGE2, hepatocyte growth factor (HGF), TGFβ, and CCL2 (82, 89). IFNγ-preconditioned MSCs also suppressed CD4^+^ and CD 8^+^ T cell and NK cell proliferation and polarized macrophages to an M2 phenotype (87, 90). However, there are no studies concerning the osteogenic ability of IFNγ-preconditioned MSCs.

**Table 1:** Immunomodulation for bone healing by the preconditioning of MSCs with pro-inflammatory cytokines *in vitro*.

**Preconditioning of MSCs with pro-inflammatory cytokines**				**Results**
**Stimulus**	**Duration**	**Species**	**Type of MSCs**	
TNFα	3 days	Human	AT-MSCs	TNFα-preconditioned human AT-MSCs (83) and their exosomes (84) promoted the proliferation and osteogenic differentiation of human primary osteoblastic cells.
TNFα	3 days	Human	AT-MSCs	TNFα-preconditioned human AT-MSCs reduced collagen type I gene expression but increased their proliferation and ALP activity (85).
TNFα	3 days	Human	BM-MSCs	TNFα-preconditioned human BM-MSCs increased their ALP activity and mineralization (86).
TNFα + LPS	3 days	Murine	BM-MSCs	A combination of TNFα and LPS enhanced osteogenic differentiation including ALP activity and matrix mineralization; however, TNFα alone or a combination (TNFα and IFNγ) did not promote osteogenesis (87).
IL6	3 days	Human	AT-MSCs	IL6-preconditioned human AT-MSCs stimulated ALP activity and mineralization (88).
IL8	3 days	Human	AT-MSCs	IL8-preconditioned human AT-MSCs did not affect proliferation or osteogenic gene expression but reduced bone nodule formation (85).
IL17F	3 days	Human	AT-MSCs	IL17F-preconditioned human AT-MSCs decreased their proliferation but enhanced ALP activity (85).

*AT-MSCs, adipose tissue-derived mesenchymal stromal cells; BM-MSCs, bone marrow-derived mesenchymal stromal cells*.

TNFα preconditioning also promotes immunoregulatory mediators such as PGE2, IDO, and HGF, but this is less pronounced compared to IFNγ preconditioning (82, 89). TNFα-preconditioned human adipose tissue-derived MSCs (AT-MSCs) (83) and their exosomes (84) promoted the proliferation and osteogenic differentiation of human primary osteoblastic cells. Another study demonstrated that TNFα-preconditioned human AT-MSCs reduced collagen type I gene expression but increased proliferation and ALP activity (85). Similarly, preconditioned human BM-MSCs exposed to TNFα enhanced their osteogenic capacity (86). However, our previous *in vitro* study demonstrated that preconditioning of murine BM-MSCs using TNFα alone or a combination TNFα and IFNγ did not promote osteogenesis; however, a combination of TNFα and LPS enhanced osteogenic differentiation including ALP activity and matrix mineralization (87).

IL17A-preconditioned MSCs increased IL6 and regulatory T cell generation and inhibited Th1 cytokine secretion (TNFα, IFNγ, IL2, and IL10) (91). In a rat calvarial defect model, the direct application of IL17A inhibited osteoblast precursor cells and bone regeneration (92). In other studies, IL17A promoted osteoblastic differentiation (46, 93, 94) and inhibited adipogenic differentiation (95) in human BM-MSCs. Ono et al. demonstrated that IL17A accelerated osteoblastogenesis *in vitro* and *in vivo* in mice (45). A recent study demonstrated that IL17 stimulated the osteogenic differentiation of the murine BM-MSC niche by using IL6 and IL1β signaling to activate ERK1/2, STAT3, and AKT (47). When these BM-MSCs were exposed to IL17 in 3D coculture with osteocytes, the BM-MSCs showed enhanced osteogenesis. However, studies concerning the osteogenic ability of IL17A-preconditioned MSCs were not reported.

The effects of preconditioning of MSCs by other pro-inflammatory cytokines such as IL6, IL8, or IL17F have been reported. IL6-preconditioned human AT-MSCs demonstrated increased ALP activity and mineralization (85, 88). IL8-preconditioned human AT-MSCs did not show changes in proliferation or osteogenic gene expression, but had reduced bone nodule formation (85). IL17F-preconditioned human AT-MSCs had decreased proliferation but enhanced ALP activity (85).

These *in vitro* studies suggest that the osteogenic ability of preconditioned MSCs may be influenced not only by pro-inflammatory cytokines but also by the species selected and the tissue of origin. Interestingly, there are few *in vivo* studies concerning the efficacy of preconditioned MSCs using pro-inflammatory cytokines; further study is needed in this area.

### Preconditioned MSC With Hypoxia

The effects of hypoxia-preconditioning on the immunomodulatory and osteogenic capacities of MSCs have become increasingly relevant, particularly as the potential therapeutic conditions of MSCs have an oxygen tension between 1 and 11%, compared to normal ambient oxygen tension (21% O_2_) (82, 89). Downstream signaling of hypoxia-inducible factors (HIFs) modulates VEGF expression and activation of SDF1 and CXCR4, thus implicating hypoxia as a modifiable element to increase MSC migration and bone healing (96). The HIF1α pathway is tightly linked to skeletal development and bone repair; mice exposed to hypoxic conditions had increased vascularity and bone healing via HIF1α and VEGF mediated pathways (97). MSCs cultured under hypoxic conditions maintain or increase their proliferation rates with increased secretion of growth factors, including VEGF, basic FGF, and PDGF-BB (98–100). In addition, increased lactate production by MSCs under hypoxic conditions could contribute to the polarization of macrophages to an anti-inflammatory M2 phenotype (101).

A recent systematic review paper (102) described that MSCs cultured under severe hypoxic conditions (<2% O_2_) with long-term exposure increased their proliferation rate and inhibited osteogenic differentiation, whereas MSCs cultured under moderate hypoxic conditions (2–5% O_2_) with short-term or cyclic exposure had accelerated osteogenic differentiation and inhibited osteoclast function *in vitro*.

Although *in vivo* applications of hypoxia-preconditioned MSCs for bone healing are limited (Table 2), Lee et al. studied human BM-MSCs that were expanded under hypoxia with 1% O_2_ and seeded on hydroxyapatite (HA)/tricalcium phosphate (TCP)-based scaffolds. These hypoxia-preconditioned BM-MSCs/HA/TCP-based scaffolds increased collagen tissue formation in a subcutaneous transplantation model in mice (103). A 21-months-old aged male rat model of hypoxia-preconditioned BM-MSCs with dimethyloxalylglycine (DMOG), a prolyl hydroxylase inhibitor, under 1% O_2_ conditions improved the repair of a critical-sized mandibular defect (106). The intramuscular injection of hypoxia-preconditioned human BM-MSCs under 1% O_2_ for 48 h exhibited markedly increased cell survival over 4 weeks in mice (107). A spheroid of hypoxia-preconditioned human BM-MSCs were cultured under 1% O_2_ for 3 days (105). Hypoxia-preconditioned BM-MSC spheroids had high osteogenic differentiation and VEGF secretion *in vitro*, and an alginate hydrogel containing hypoxia-preconditioned BM-MSC spheroids improved bone healing of a critical-sized femoral bone defect in male athymic rats at the age of 10–12 weeks. Thus, although the molecular mechanisms of hypoxic conditioning on MSCs remain unclear, these results demonstrate the translatability of biochemical and *in vitro* studies of MSC hypoxia to *in vivo* therapies, even under challenging conditions such as critical-sized defects in aged models.

**Table 2:** Immunomodulation for bone healing by hypoxia-reconditioned MSC *in vivo*.

**Hypoxia-preconditioning**				**Study models**	**Carriers of cells**	**Results**
**Hypoxic condition**	**Duration**	**Species**	**Type of MSCs**			
1% O_2_	-	Human	BM-MSCs	A subcutaneous transplantation model in immunocompromised mice	HA/TCP scaffolds	Hypoxia-preconditioned MSCs/HA/TCP scaffolds had increased bone tissue formation, as measured by histological analysis (103).
1% O_2_	96 h+	Rat	BM-MSCs	A critical-sized mandibular defect model in 21-months-old aged male *SD* rats	Gelatin sponges	Hypoxia-preconditioned BM-MSCs/gelatin sponges showed accelerated angiogenesis and osteogenesis and improved bone healing as measured by microCT and histological analyses (104).
1% O_2_	3 days	Human	BM-MSCs	A critical-sized femoral bone defect model in male athymic rats (10–12 weeks old)	Alginate hydrogels	Hypoxia-preconditioned MSC spheroids/alginate hydrogels accelerated angiogenesis at 2 weeks, and improved bone healing and mechanical strength at 12 weeks, as measured by microCT, mechanical testing, and histological analysis (105).

*BM-MSC. bone marrow-derived mesenchymal stromal cells; HA, hydroxyapatite; TCP, tricalcium phosphate; DMOG, dimethyloxalylglycine*.

### Immunomodulation by Accelerating the Resolution of Inflammation via Local Delivery of Anti-inflammatory Cytokines

Direct application of anti-inflammatory cytokines can modify the microenvironment and promote bone healing, particularly when consideration is given to the timing and delivery method (Table 3). Anti-inflammatory cytokines IL4 and IL13 inhibit the proliferation of human osteoblasts (115) but increase collagen secretion, ALP expression, and mineralization (116). Alternatively, IL4 decreased osteogenic differentiation in monoculture of mouse BM-MSCs (108) and human AT-MSCs (88). However, IL4 and IL13 stimulate the polarization of macrophages from the inflammatory M1 phenotype to the anti-inflammatory M2 phenotype (117), and crosstalk between MSCs and macrophages is critical for successful bone healing (5). Therefore, monoculture models may not accurately reflect the full immunomodulatory and osteogenic potential of anti-inflammatory cytokines *in vivo*. An MSC-macrophage coculture model using either primary murine BM-MSCs (55) or pre-osteoblastic MC3T3 cells (109) with M1 macrophages showed enhanced bone mineralization and ALP activity. Moreover, the addition of IL4 at 72 h to polarize the M1 macrophages to an M2 phenotype further increased calcified matrix formation, compared to introducing IL4 earlier (109, 110). Thus, acute inflammation is necessary to initiate bone healing; the specific timing of the resolution of inflammation is critical for optimal bone formation *in vitro*.

**Table 3:** Immunomodulation for bone healing by the resolution of inflammation with anti-inflammatory cytokines.

**Anti-inflammatory cytokines**	**Studies**	**Models**	**Delivery methods**	**Results**
IL4	*In vitro*	Monoculture	-	IL4 decreased osteogenic differentiation in monoculture of mouse BM-MSCs (108), human AT-MSCs (88).
IL4	*In vitro*	MSC-macrophage coculture	-	IL4 enhanced bone mineralization and ALP activity of murine MSCs (55) or pre-osteoblastic MC3T3 cells (109) in coculture with M1 macrophages.
IL4	*In vitro*	MSC-macrophage coculture	-	The addition of IL4 at 72 h to polarize the M1 macrophages to an M2 phenotype further increased calcified matrix formation, compared to introducing IL4 earlier (110).
IL4, IL13	*In vivo*	A murine bone defect model	An IL4 and IL13 loaded collagen scaffolds	A collagen scaffold containing IL4 and IL13 increased callus formation in an *in vivo* murine bone defect model (111).
IL4	*In vivo*	A rat calvarial defect model	Daily injection of IL4 into the scaffold from day 3 to day 7	Low dose (10 ng) IL4 significantly increased bone formation and vascularization, with favorable M1/M2 polarization ratios, compared to higher doses (50 ng and 100 ng) of IL4 or none (112).
IL4	*In vivo*	A murine distal femoral bone marrow cavity model	Genetically modified IL4 secreting MSCs	Genetically modified IL4 secreting MSCs injected into the murine distal femoral bone marrow cavity increased bone mineralization (113).
IL4	*In vivo*	A mouse calvarial model with PE	Daily local IL4 injection	Bone loss was significantly decreased following IL4 administration to PE treated calvaria; increased M1/M2 ratio in the PE treated calvaria, which decreased with addition of IL4 (114).
IL4	*In vivo*	A mouse continuous PE femoral intramedullary infusion model	IL4 was infused into mouse distal femurs by osmotic pump	Continuous local IL4 delivery was an effective means to prevent particle-induced bone loss and enhance bone structural properties in the context of wear particle-induced inflammation (72).

*AT-MSCs, adipose tissue-derived mesenchymal stromal cells; BM-MSCs, bone marrow-derived mesenchymal stromal cells; PE, polyethylene particle*.

Others have demonstrated that a collagen scaffold containing IL4 and IL13 increased callus formation in an *in vivo* murine bone defect model (111). Zheng et al. (112) implanted a decellularized bone matrix (DBM) scaffold into a calvarial defect in rats and performed daily injection of different IL4 doses (0, 10, 50, and 100 ng) through the skin directly over the scaffold from day 3 to 7 after surgery. They found that a rat cranial bone defect model with low dose (10 ng) IL4 loaded into decellularized bone matrix significantly increased bone formation and vascularization, with favorable M1/M2 polarization ratios, compared to higher doses (50 and 100 ng) of IL4 or matrix alone. Indeed, as immunomodulation to improve bone healing becomes a therapeutic strategy, immunomodulatory scaffolds are becoming more complex. Other studies have fabricated a functional scaffold with differential release of immunomodulatory molecules, such as a decellularized bone scaffold with sustained release of IL4 via biotin-streptavidin binding (118) and a collagen scaffold with poly(lactic-co-glycolic acid)-multistage silicon particles composite microspheres releasing IL4 (119). A more recent method to provide controlled, direct release of anti-inflammatory cytokines is through cell modification.

Our group (108) has established two types of genetically modified IL-4 secreting BM-MSCs using lentiviral vectors: one is a continuously-IL4-overexpressing BM-MSC driven by the cytomegalovirus (CMV) promoter, and the other is an NFκB-sensing-IL4-overexpressing BM-MSC driven by the NFκB promoter. NFκB-sensing-IL4-overexpressing BM-MSCs produce IL4 when NFκB is activated; thus, these cells secrete IL4 under the conditions of an inflammatory stimulus only. Transplantation of both types of genetically modified IL-4 secreting BM-MSCs into the murine distal femoral bone marrow cavity increased bone mineralization (113).

Although these *in vivo* studies demonstrated the therapeutic effect of IL4 and IL13 for bone healing, a comprehensive comparison of the optimal dose and delivery timing and methods (e.g., biomaterials and genetically modified MSCs, etc.) of pro-inflammatory cytokines for bone healing is still lacking; further *in vivo* studies are needed.

IL10 is another potent anti-inflammatory cytokine that can affect bone formation. Mechanistic studies in mice found that IL10 promotes chondrocyte proliferation and differentiation via the bone morphogenetic protein (BMP) pathway, thus influencing endochondral bone formation (120). IL10 deficient mice showed suppressed bone formation and osteoblastogenesis, resulting in osteopenia and increased bone fragility (121, 122). However, various concentrations of IL10 exert different effects on the osteogenesis of human BM-MSCs. Low physiologic concentrations of IL10 (0.01–1.0 ng/ml) activate the p38/MAPK signaling pathway to promote osteogenesis, whereas higher doses of IL10 (10–100 ng/ml) inhibit p38/MAPK signaling by activating NF-kB, inhibiting osteogenesis (123). An *in vitro* osteoblast-osteoclast coculture model demonstrated that genetically-modified IL10 and TGFβ overexpressing osteoclasts inhibited osteoblast apoptosis and decreased osteoclast formation and bone absorption ability (124). Further translational studies using IL10 and other immunomodulatory molecules are needed.

Immunomodulatory strategies to resolve chronic inflammatory bone disease apply these same principles but require more advanced models and the ability to respond to inflammatory stimuli. For example, in wear particle-induced chronic inflammation, *in vivo* studies using a mouse calvarial model (114) and continuous polyethylene particle femoral intramedullary infusion model (125) showed that IL4 prevented bone loss and accelerated bone formation by modulating local macrophage polarization to an M2 phenotype. In a recent MSC-macrophage coculture study simulating wear particle-induced inflammation, we demonstrated that NFκB-sensing-IL4-overexpressing BM-MSCs decreased ALP activity and osteocalcin expression (early osteogenic markers) but increased mineralization using Alizarin red staining (late osteogenic marker) (126). These results suggest that NFκB-sensing-IL4-overexpressing BM-MSCs are useful to enhance osteogenesis at a later stage. In combination with preconditioned BM-MSCs, which increased early osteogenic markers, these immunomodulatory strategies may increase bone regeneration at different stages of chronic bone inflammatory disease.

## Discussion

This review summarizes current fundamental knowledge underlining the importance of acute inflammation for normal bone healing after injury. Numerous studies have now substantiated that deficiencies in critical cell crosstalk, inhibition of the natural processes of acute inflammation and its resolution, or chronic inflammation due to a persistent adverse stimulus can lead to impaired fracture healing. Thus, an initial and optimal transient stage of acute inflammation is one of the crucial events during fracture healing.

**Figure 3:**
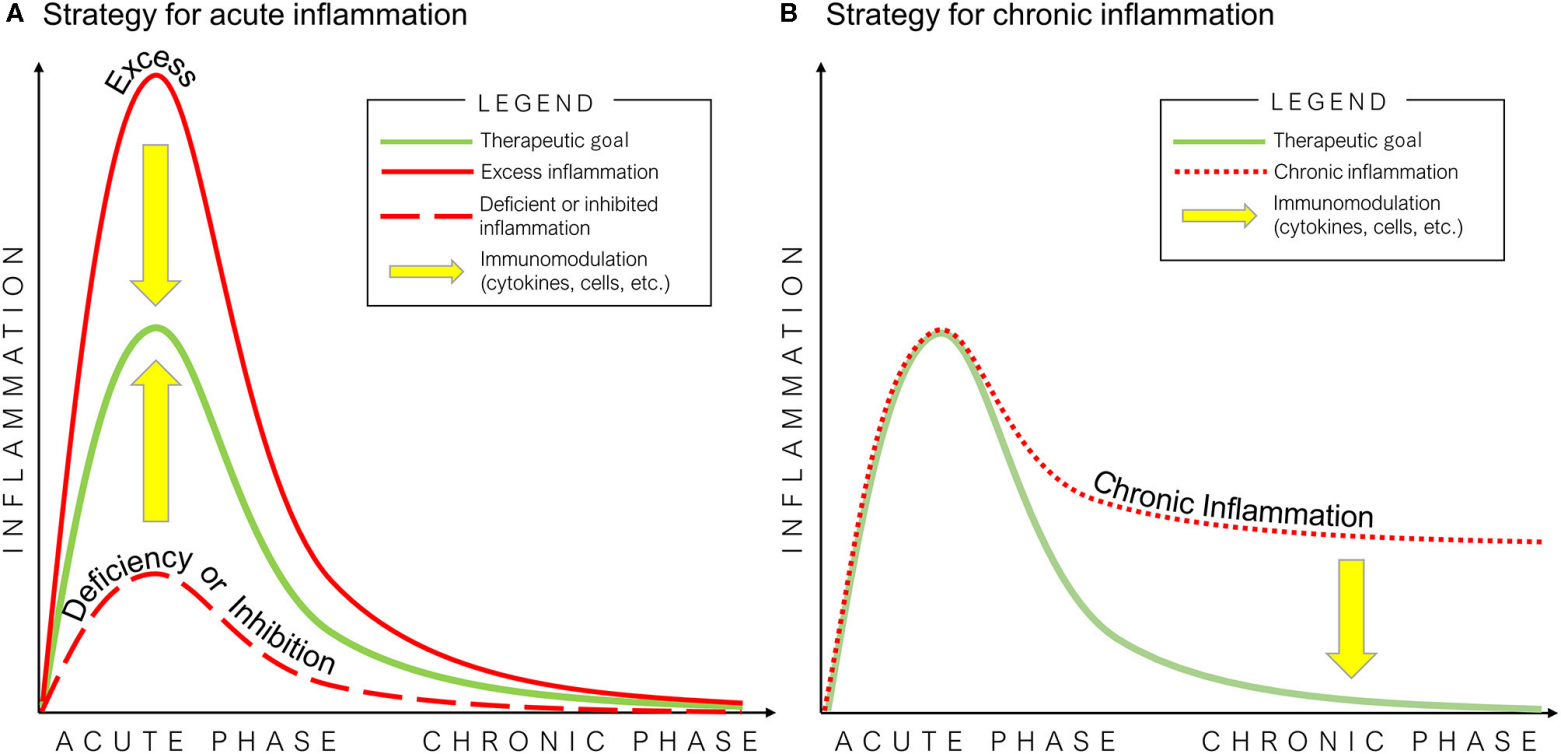
Potential strategies of immunomodulation for bone healing in acute **(A)** and chronic **(B)** inflammation. The optimal inflammatory process for bone healing is mediated by an initial and optimal transient stage of acute inflammation, followed by the resolution of inflammation (green line). Excess (solid red line) or deficiency/inhibition of acute inflammation (dashed red line), and chronic inflammation (dotted red line) interfere with the healing of bone defects.

Once the basic principles associated with normal bone healing are clearly understood, then potential strategies for immunomodulation of critical biological events may be exploited (Figure 3). For example, *in vitro* studies demonstrated that preconditioning of MSCs by pro-inflammatory cytokines or hypoxic conditions enhances their osteogenic potential. However, a comprehensive comparison of the osteogenic ability of MSCs derived from different tissue sources is still limited. In addition, no study has directly and comprehensively compared preconditioning of MSCs using different pro-inflammatory cytokine combinations vs. hypoxic conditions. Furthermore, there are few *in vivo* studies concerning the efficacy of preconditioned MSCs. Thus, the clinical application of preconditioned MSCs and other novel technologies is not fully known. Further *in vivo* translational studies are needed in this regard. *In vitro* studies concerning immunomodulation for the resolution of inflammation using anti-inflammatory cytokines also demonstrated their therapeutic potential to improve bone healing. The clinical application of immunomodulation for bone healing using anti-inflammatory cytokines would not be suitable before 3 days from the onset of acute inflammation. However, the optimal dose, timing, and methods of delivery of pro-inflammatory cytokines have not been fully clarified. Furthermore, similar to preconditioned MSCs, *in vivo* translational studies concerning the efficacy of immunomodulation for inflammatory resolution for bone healing are limited. Therefore, the therapeutic effects of immunomodulation for bone healing using preconditioned MSCs, anti-inflammatory cytokines to suppress inflammation, or a combination of these strategies should be further evaluated in future *in vivo* translational studies.

## Author Contributions

All authors contributed to the initial concepts and writing of the present manuscript.

## Conflict of Interest

The authors declare that the research was conducted in the absence of any commercial or financial relationships that could be construed as a potential conflict of interest.
